# Enhancing the Drug Release and Physicochemical Properties of Rivaroxaban via Cyclodextrin Complexation: A Comprehensive Analytical Approach

**DOI:** 10.3390/ph18060761

**Published:** 2025-05-22

**Authors:** Cristina Solomon, Valentina Anuța, Iulian Sarbu, Emma Adriana Ozon, Adina Magdalena Musuc, Veronica Bratan, Adriana Rusu, Vasile-Adrian Surdu, Cătălin Croitoru, Abhay Chandak, Roxana Mariuca Gavriloaia, Teodora Dalila Balaci, Denisa Teodora Niță, Mirela Adriana Mitu

**Affiliations:** 1 Faculty of Pharmacy, “Carol Davila” University of Medicine and Pharmacy, 6 Traian Vuia St., 020956 Bucharest, Romania; cristina.solomon@drd.umfcd.ro (C.S.); teodora.balaci@umfcd.ro (T.D.B.); denisa-teodora.nita0720@stud.umfcd.ro (D.T.N.); mirela.mitu@umfcd.ro (M.A.M.); 2 Innovative Therapeutic Structures Research and Development Centre (InnoTher), “Carol Davila” University of Medicine and Pharmacy, 6 Traian Vuia Street, 020956 Bucharest, Romania; valentina.anuta@umfcd.ro; 3 Faculty of Pharmacy, “Titu Maiorescu” University, 004051 Bucharest, Romania; iulian.sarbu@prof.utm.ro (I.S.); roxana.gavriloaia@prof.utm.ro (R.M.G.); 4 Institute of Physical Chemistry—Ilie Murgulescu, Romanian Academy, 060021 Bucharest, Romania; vbratan@icf.ro (V.B.); arusu@icf.ro (A.R.); 5 Department of Materials Science, Faculty of Materials Science and Engineering, Transilvania University of Brasov, 29 Eroilor Blvd., 500036 Brasov, Romania; vasile.surdu@unitbv.ro; 6 Materials Engineering and Welding Department, Transilvania University of Brasov, 500036 Brasov, Romania; c.croitoru@unitbv.ro; 7 Zentiva Group, U Kabelovny 529/16, 102 00 Praha-Dolní Měcholupy, Czech Republic; abhaykumar.chandak@zentiva.com

**Keywords:** rivaroxaban, β-cyclodextrin, methyl-β-cyclodextrin, hydroxypropyl-β-cyclodextrin, solubility enhancement, pharmaceutical formulation, drug release

## Abstract

**Background/Objectives**: Rivaroxaban, an oral anticoagulant, shows poor aqueous solubility, posing significant challenges to its bioavailability and therapeutic efficiency. The present study investigates the improvement of rivaroxaban’s solubility through the formation of different inclusion complexes with three cyclodextrin derivatives, such as β-cyclodextrin (β-CD), methyl-β-cyclodextrin (Me-β-CD), and hydroxypropyl-β-cyclodextrin (HP-β-CD) prepared by lyophilization in order to stabilize the complexes and improve dissolution characteristics of rivaroxaban. **Methods**: The physicochemical properties of the individual compounds and the three lyophilized complexes were analysed using Fourier transform infrared spectroscopy (FTIR), scanning electron microscopy (SEM), X-ray diffraction (XRD), and thermogravimetric analysis (TGA). **Results**: FTIR spectra confirmed the formation of non-covalent interactions between rivaroxaban and the cyclodextrins, suggesting successful encapsulation into cyclodextrin cavity. SEM images revealed a significant morphological transformation from the crystalline structure of pure rivaroxaban and cyclodextrins morphologies to a more porous and amorphous matrix in all lyophilized complexes. XRD patterns indicated a noticeable reduction in drug crystallinity, supporting enhanced potential of the drug solubility. TGA analysis demonstrated improved thermal stability in the inclusion complexes compared to the individual drug and cyclodextrins. Pharmacotechnical evaluation revealed that the obtained formulations (by comparison with physical mixtures formulations) possessed favorable bulk and tapped density values, suitable compressibility index, and good flow properties, making all suitable for direct compression into solid dosage forms. **Conclusions**: The improved cyclodextrins formulation characteristics, combined with enhanced dissolution profiles of rivaroxaban comparable to commercial Xarelto^®^ 10 mg, highlight the potential of both cyclodextrin inclusion and lyophilization technique as synergistic strategies for enhancing the solubility and drug release of rivaroxaban.

## 1. Introduction

The solubility of poorly water-soluble drugs poses a significant challenge in the pharmaceutical industry, particularly for drugs with low bioavailability, as it directly affects their dissolution rate, absorption, and therapeutic efficacy. Rivaroxaban, a widely used direct oral anticoagulant (DOAC) and a direct Factor Xa inhibitor, is effective in preventing and treating thromboembolic disorders [1,2]. However, its relatively low solubility in aqueous media limits its absorption in the gastrointestinal tract, leading to unpredictable bioavailability and potential variability in medical treatment [3]. Consequently, enhancing the solubility of rivaroxaban is critical to optimizing its medical efficiency and to ensuring a consistent healing reaction.

Cyclodextrins (CDs), a family of cyclic oligosaccharides, have been developed currently in the pharmaceutical industry with a significant increasing use as a promising approach to improve the solubility of poorly water-soluble drugs [4]. These molecules possess a hydrophilic outer surface and a hydrophobic central cavity, which allows them to form inclusion complexes with hydrophobic drugs [5]. By encapsulating the drug molecules within their cavity, cyclodextrins can significantly improve the solubility, stability, and dissolution characteristics of these compounds. Among the various cyclodextrins, β-cyclodextrin (β-CD) and its modified derivatives, such as methyl-β-cyclodextrin (Me-β-CD) and hydroxypropyl-β-cyclodextrin (HP-β-CD), are particularly significant due to their enhanced solubility, lower toxicity, and ability to form stable inclusion complexes with hydrophobic drugs like rivaroxaban.

In addition to cyclodextrin complexation, the technique of lyophilization (freeze-drying) offers considerable benefits for improving the dissolution and stability of drug formulations [6,7]. By decreasing the moisture content from the drug-cyclodextrin complex under low temperatures, lyophilization preserves the integrity of the complex and transforms it into an amorphous, highly soluble form [8]. This process not only enhances the solubility of the drug but also improves the long-term stability of the formulation by reducing degradation and maintaining a stable structure over time.

Despite numerous studies on cyclodextrin-based drug formulations, there is a lack of comprehensive research comparing different cyclodextrin derivatives, particularly in the case of rivaroxaban. Most existing studies focus on individual cyclodextrin complexes without providing a comparative analysis of their effects on solubility enhancement and physicochemical properties. Additionally, while lyophilization has been widely used to stabilize cyclodextrin-based complexes, few studies have explored its combined effect with different cyclodextrin derivatives to optimize the properties of rivaroxaban [9]. This study aims to evaluate the potential of several cyclodextrin derivatives, such as β-cyclodextrin (β-CD), methyl-β-cyclodextrin (Me-β-CD), and hydroxypropyl-β-cyclodextrin (HP-β-CD), in enhancing the solubility and dissolution profile of rivaroxaban through the formation of inclusion complexes by the lyophilization technique. The present research goal is to investigate the physicochemical properties of these three inclusion complexes using different and complementary analytical techniques, including Fourier transform infrared spectroscopy (FTIR), scanning electron microscopy (SEM), X-ray diffraction (XRD), and thermogravimetric analysis (TGA). These techniques provide insights into the molecular interactions, structural modifications, and stability of the obtained inclusion complexes. The novelty of this study lies in its comprehensive comparison of several cyclodextrin derivatives in the formulation of rivaroxaban inclusion complexes. By exploring how each cyclodextrin derivative influences the solubility, dissolution rate, and stability of rivaroxaban, the study provides a more detailed understanding of their potential in drug formulation. This study attempts to evaluate the synergistic effect of cyclodextrin complexation and lyophilization on rivaroxaban, due to insufficient development in the literature and pharmaceutical domain. The results of this study can contribute to the development of optimized rivaroxaban formulations that enhance its release and therapeutic efficacy, providing significant improvements in anticoagulation therapy.

## 2. Results and Discussions

### 2.1. Phase-Solubility Diagrams

**Figure 1:**
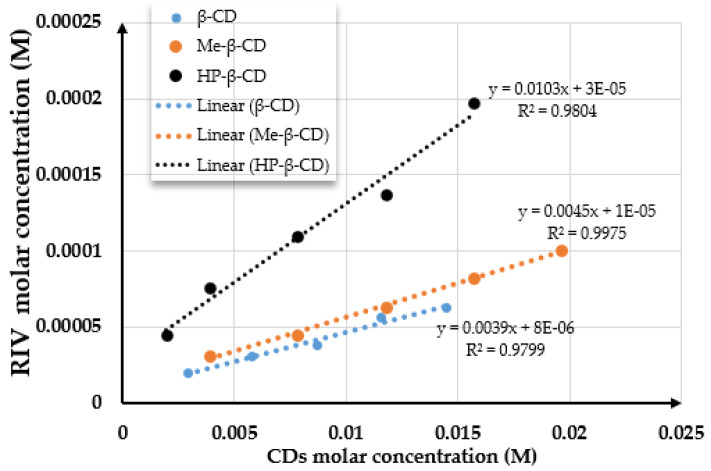
The phase-solubility diagram of rivaroxaban–β-CD, rivaroxaban–Me-β-CD, and rivaroxaban–HP-β-CD systems.

Based on the classification established by Higuchi and Connors, the phase-solubility diagrams obtained for the rivaroxaban–β-CD, rivaroxaban–Me-β-CD, and rivaroxaban–HP-β-CD systems (Figure 1) exhibited an A_L_-type profile for all three compounds, indicative of the formation of water-soluble inclusion complexes. A_L_-type diagrams are characterized by a linear increase in the apparent solubility of the active pharmaceutical ingredient with rising cyclodextrin concentration across the studied range. The linearity of the plot and the calculated regression parameters (with a slope less than 1) suggest the formation of a 1:1 stoichiometric inclusion complex between rivaroxaban and β-CD, Me-β-CD, and HP-β-CD in aqueous medium.

Table 1 shows the regression parameters and the apparent complexation constant (*K*_st_) for the rivaroxaban–β-CD, rivaroxaban–Me-β-CD, and rivaroxaban–HP-β-CD systems.

The value of the intrinsic solubility of rivaroxaban in the absence of cyclodextrins (*S*_0_), calculated as the average of the intrinsic solubilities, was 0.000016 M. The phase solubility diagrams of rivaroxaban with β-CD, Me-β-CD, and HP-β-CD are shown in Figure 1.

The calculated stability constant (*K*_st_) values for the rivaroxaban–cyclodextrin inclusion complexes show the general trend *K*_st_(HP-β-CD) > *K*_st_(Me-β-CD) > *K*_st_(β-CD). These values indicate that the complexes are sufficiently stable to enhance aqueous solubility and improve drug dissolution. Specifically, the observed *K*_ₛₜ_ values suggest the formation of a stable 1:1 inclusion complex.

### 2.2. Binary Systems Characterization

The lyophilized compounds were white, uniform, and amorphous powders, while the physical mixtures were also white, but fine, crystalline powders.

### 2.3. Physicochemical Characterization

**Figure 2:**
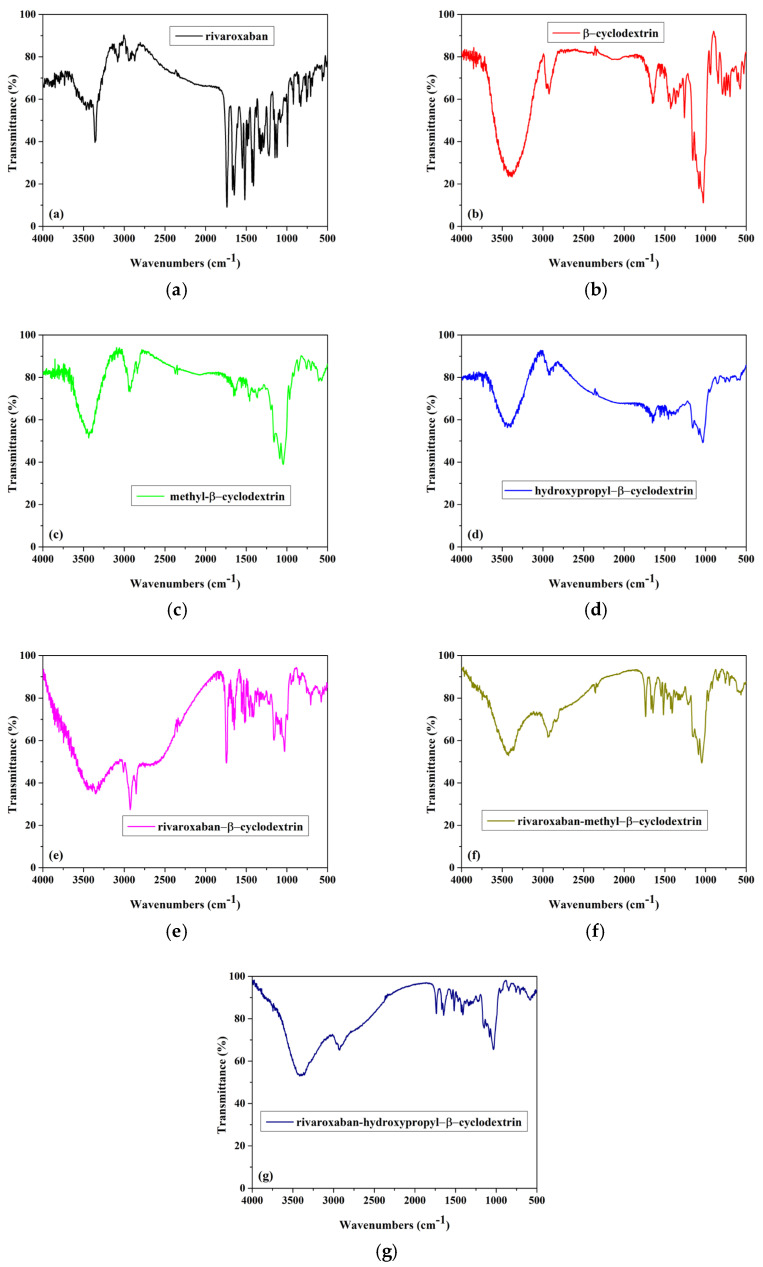
FTIR spectra of (**a**) rivaroxaban, (**b**) β-cyclodextrin, (**c**) methyl-β-cyclodextrin, (**d**) hydroxypropyl-β-cyclodextrin, (**e**) inclusion complex of rivaroxaban-β-cyclodextrin, (**f**) inclusion complex of rivaroxaban-methyl-β-cyclodextrin, and (**g**) inclusion complex of rivaroxaban-hydroxypropyl-β-cyclodextrin.

*FTIR analysis*. Figure 2 shows the FTIR spectra recorded for (a) rivaroxaban, (b) β-cyclodextrin, (c) methyl-β-cyclodextrin, (d) hydroxypropyl-β-cyclodextrin, (e) inclusion complex of rivaroxaban-β-cyclodextrin, (f) inclusion complex of rivaroxaban-methyl-β-cyclodextrin, and (g) inclusion complex of rivaroxaban-hydroxypropyl-β-cyclodextrin.

The FTIR spectrum of rivaroxaban (Figure 2a) revealed several characteristic bands: the N-H stretch from the secondary amide group appears at 3358 cm^−1^, the bands at 1646 cm^−1^ are attributed to the stretching frequencies from the amide group, and the band from 1735 cm^−1^ is attributed to C=O carbonyl stretch from the amide group. The bands from the region 2800–3000 cm^−1^ are attributed to C-H stretching vibrations. Because the characteristic peak of the rivaroxaban form I appeared at 1146 cm^−1^ (as was reported in the literature), even a slight difference in the wavenumbers is observed compared with the literature; it can be assumed that the rivaroxaban spectrum is for the rivaroxaban form I [10,11].

The FTIR spectra of β-cyclodextrin, methyl-β-cyclodextrin, and hydroxypropyl-β-cyclodextrin are presented in Figure 2b–d. The following bands, characteristics to cyclodextrin compounds, are observed.

Figure 2b—The FTIR spectrum of β-CD (Figure 2b) shows a large band in the range of 3000–3600 cm^−1^, corresponding to the strong O–H stretching vibrations; the band at 2925 cm^−1^ is assigned to the symmetric stretching of the C-H bonds in the CH and CH_2_ groups. Additionally, the strong band at 1029 cm^−1^ is attributed to the O–H bending vibration.

Figure 2c—The FTIR spectrum of Me-β-CD (Figure 2c) exhibits the primary characteristic bands associated with cyclodextrin compounds. The peak at 3428 cm^−1^ is attributed to the strong O–H stretching vibrations, at 2933 cm^−1^ and 2840 cm^−1^ are observed bands which correspond to the aliphatic C-H stretching vibrations of Me-β-CD. The stretching frequency of the C-OH groups in the primary and secondary positions of the Me-β-CD molecule is observed around 1040 cm^−1^ [12].

Figure 2d—The FTIR spectrum of HP-β-CD (Figure 2d) reveals the following peaks: the broad band at 3438 cm^−1^ is attributed to the O-H stretching vibration, caused by intramolecular hydrogen bonding; the peak at 2918 cm^−1^ corresponds to the anti-symmetric stretching vibration of the methyl groups (C-H), while the band at 1653 cm^−1^ is associated with O-H bending vibrations; the peak at 1163 cm^−1^ is due to C-O vibrations [13].

The FTIR spectra of all inclusion complexes (Figure 2e–g) present changes compared with individual components. The disappearance or significant reduction of the characteristic bands of rivaroxaban in the FTIR spectra of RIV-β-CD, RIV-Me-β-CD, and RIV-HP-β-CD complexes obtained using the lyophilization method suggests strong interactions between rivaroxaban and the cyclodextrin compounds, indicating possible complexation through the inclusion of the drug into the cyclodextrin cavity. These changes observed in the FTIR spectra of all complexes, such as shifts in absorption peaks, decreased intensity, or even complete disappearance of certain peaks, are evidence of the rivaroxaban inclusion in the cyclodextrins’ cavities.

**Figure 3:**
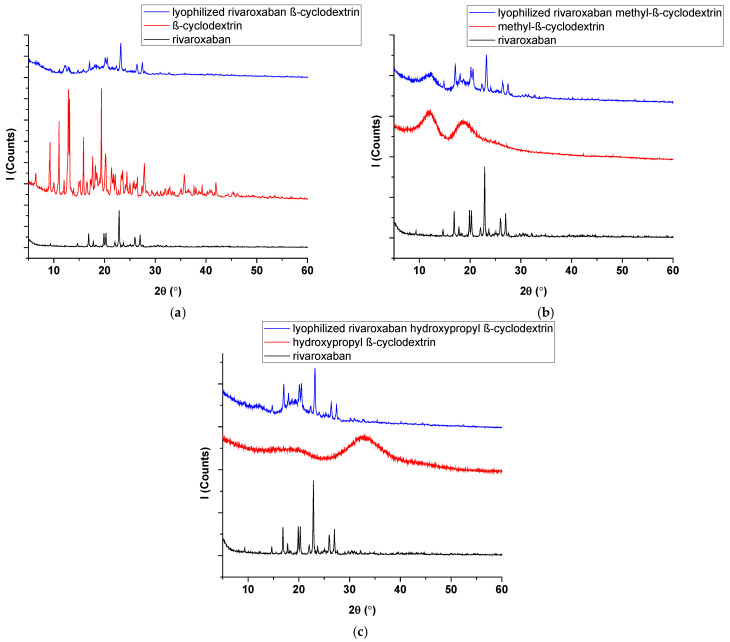
XRD diffraction spectra of (**a**) rivaroxaban, β-cyclodextrin, and rivaroxaban-β-cyclodextrin; (**b**) rivaroxaban, methyl-β-cyclodextrin, and rivaroxaban-methyl-β-cyclodextrin; (**c**) rivaroxaban, hydroxypropyl-β-cyclodextrin, and rivaroxaban-hydroxypropyl-β-cyclodextrin.

*XRD analysis*. Figure 3 shows the XRD diffraction spectra of (a) rivaroxaban, β-cyclodextrin, and rivaroxaban-β-cyclodextrin; (b) rivaroxaban, methyl-β-cyclodextrin, and rivaroxaban-methyl-β-cyclodextrin; (c) rivaroxaban, hydroxypropyl-β-cyclodextrin, and rivaroxaban-hydroxypropyl-β-cyclodextrin.

The X-ray diffractogram of rivaroxaban exhibits numerous well-defined and sharp diffraction peaks, confirming the crystalline nature of the compound [10]. The X-ray diffraction pattern of β-CD (Figure 3a) displays distinct and well-defined peaks, with characteristic diffraction angles observed at 9°, 13°, 15°, 19°, 22°, and 24° (2θ). The X-ray diffraction patterns of methyl-β-CD (Figure 3b) and hydroxypropyl-β-cyclodextrin (Figure 3c) confirm their amorphous nature with two broad peaks for both compounds. The XRD diffraction patterns for all three inclusion compounds displayed well-defined peaks but with lower intensity. The absence of characteristic diffraction peaks of rivaroxaban in the XRD pattern indicates a strong interaction between the drug and cyclodextrins, supporting the formation of an inclusion complex, as was confirmed by FTIR analysis.

**Figure 4:**
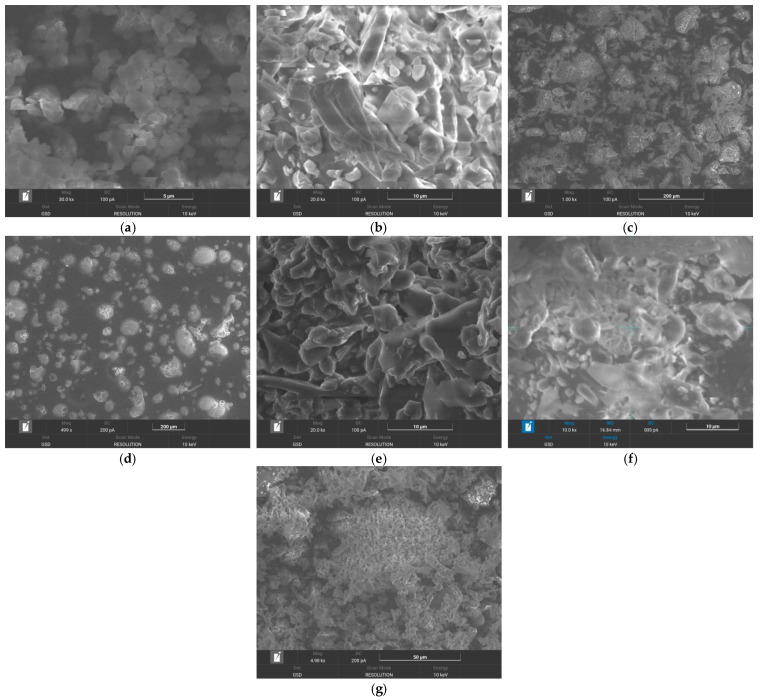
SEM images of (**a**) rivaroxaban, (**b**) β-cyclodextrin, (**c**) methyl-β-cyclodextrin, (**d**) hydroxypropyl-β-cyclodextrin, (**e**) inclusion complex of rivaroxaban-β-cyclodextrin, (**f**) inclusion complex of rivaroxaban-methyl-β-cyclodextrin, and (**g**) inclusion complex of rivaroxaban-hydroxypropyl-β-cyclodextrin.

*SEM analysis.* The SEM images of (a) rivaroxaban, (b) β-cyclodextrin, (c) methyl-β-cyclodextrin, (d) hydroxypropyl-β-cyclodextrin, (e) inclusion complex of rivaroxaban-β-cyclodextrin, (f) inclusion complex of rivaroxaban-methyl-β-cyclodextrin, and (g) inclusion complex of rivaroxaban-hydroxypropyl-β-cyclodextrin are presented in Figure 4.

Rivaroxaban (Figure 4a) is characterized by irregular-shaped crystals [14]. The SEM image of β-cyclodextrin (Figure 4b) reveals uniformly distributed crystalline particles with distinct polyhedral shapes. Methyl-β-cyclodextrin (Figure 4c) and hydroxypropyl-β-cyclodextrin (Figure 4d) show an amorphous morphology, composed of spherical particles [13]. The SEM image of the inclusion complexes obtained via lyophilization reveals an amorphous morphology indicative of the formation of a new solid phase, for all complexes (Figure 4e–g). These structural transformations suggest a potential interaction between rivaroxaban and cyclodextrins, likely resulting from the inclusion of the drug within the cyclodextrin cavities.

**Figure 5:**
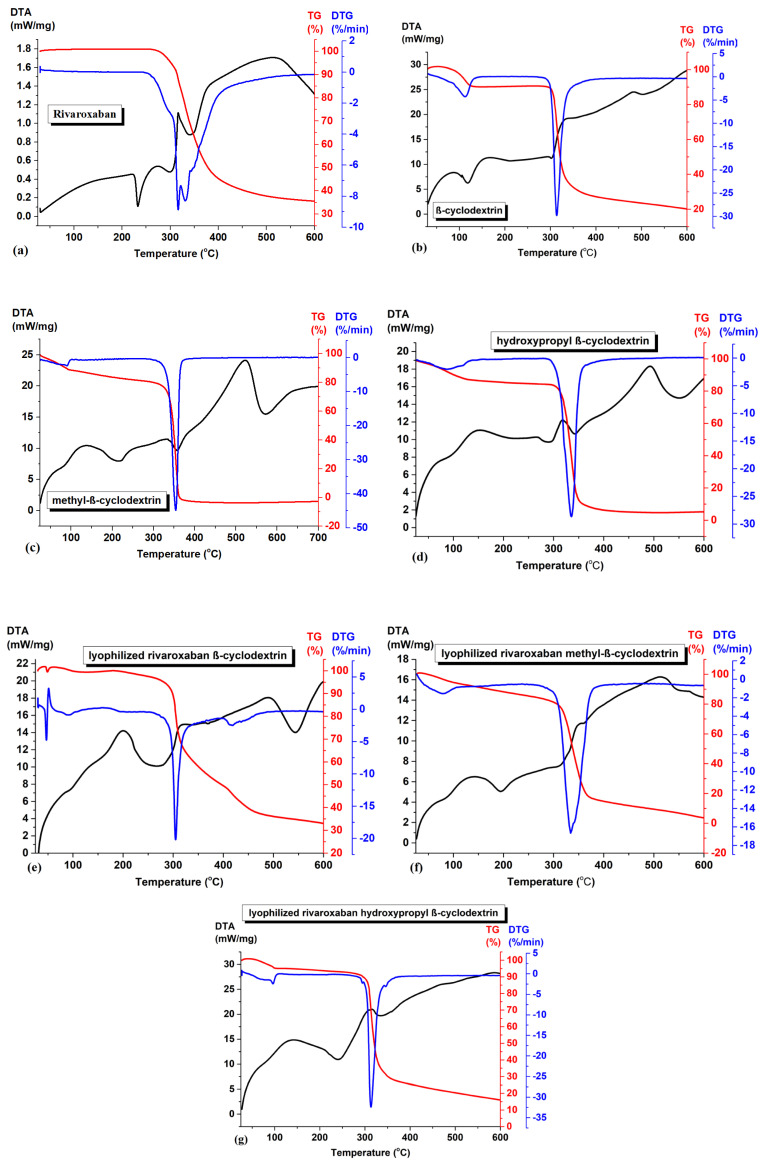
Thermal curves of (**a**) rivaroxaban, (**b**) β-cyclodextrin, (**c**) methyl-β-cyclodextrin, (**d**) hydroxypropyl-β-cyclodextrin, (**e**) inclusion complex of rivaroxaban-β-cyclodextrin, (**f**) inclusion complex of rivaroxaban-methyl-β-cyclodextrin, and (**g**) inclusion complex of rivaroxaban-hydroxypropyl-β-cyclodextrin.

*Thermal analysis*. The thermal curves (TG-DTG and DTA) of the studied compounds are represented in Figure 5. The thermal curves were obtained at 10 °C/min.

During heating in nitrogen atmosphere, after the melting process (indicated in DTA curve of rivaroxaban, Figure 5a at 232.7 °C), the TG curve shows a mass loss of 64%, in the temperature range 260–600 °C, and a residue of 36% was left at the end of the experiment at 600 °C. The decomposition process is associated with a complex mechanism (on DTG curve from Figure 5a) [15]. All TG and DTA curves of β-cyclodextrin, methyl-β-cyclodextrin, and hydroxypropyl-β-cyclodextrin show, initially, between 80–120 °C, a mass loss characterized by an endothermic process on DTA curves corresponding to the loss of absorbed water molecules from the inside and outside of the cyclodextrin cavity (9.7% for β-cyclodextrin (Figure 5b), 11.2% methyl-β-cyclodextrin (Figure 5c), and 13.4% hydroxypropyl-β-cyclodextrin (Figure 5d). For all three cyclodextrins, the degradation process occurs between 300 and 400 °C.

The disappearance of the rivaroxaban melting point in all three inclusion complexes suggests the formation of a new phase. From Figure 5e–g, several features can be noticed:

(i) The absence of the melting peak of rivaroxaban clearly signifies that the drug was completely included in the cyclodextrin cavity;
(ii) The dehydration processes have a mass loss lower than the parent cyclodextrins (0.63% for inclusion complex of rivaroxaban-β-cyclodextrin (Figure 5e); 8.18% for inclusion complex of rivaroxaban-methyl-β-cyclodextrin (Figure 5f), and 5.13% for inclusion complex of rivaroxaban-hydroxypropyl-β-cyclodextrin (Figure 5g)) suggesting that the water molecule inside the cyclodextrin cavity were replaced by rivaroxaban molecules;
(iii) The degradation processes of the inclusion complexes are not completed at a temperature around 600 °C compared with the parent cyclodextrins, suggesting a higher thermal stability of the inclusion complexes. The TGA/DTA thermograms of the inclusion complexes show the near-complete disappearance of the thermal events characteristic of the individual components, strongly indicating the formation of a new compound.

All the above-mentioned observations indicate a progressive loss of crystalline structure in the rivaroxaban and all cyclodextrin compounds, resulting from processing by the lyophilization method. This transformation is fully realized in all the inclusion complexes, which exhibit a distinct chemical and crystalline identity, as confirmed by FTIR, XRD, SEM, and TGA analyses.

### 2.4. Precompression Studies for the Tablets Containing RIV-CD Binary Systems

The materials’ particle size is a decisive factor for the filling of the die, the flowability of the powders, and the consistency and integrity of tablets when using direct compression technology. To produce tablets with acceptable pharmacotechnical and biopharmaceutical qualities, an ideal particle size distribution must be attained [16]. The particle size distribution range must be determined during the pre-compression tests.

**Figure 6:**
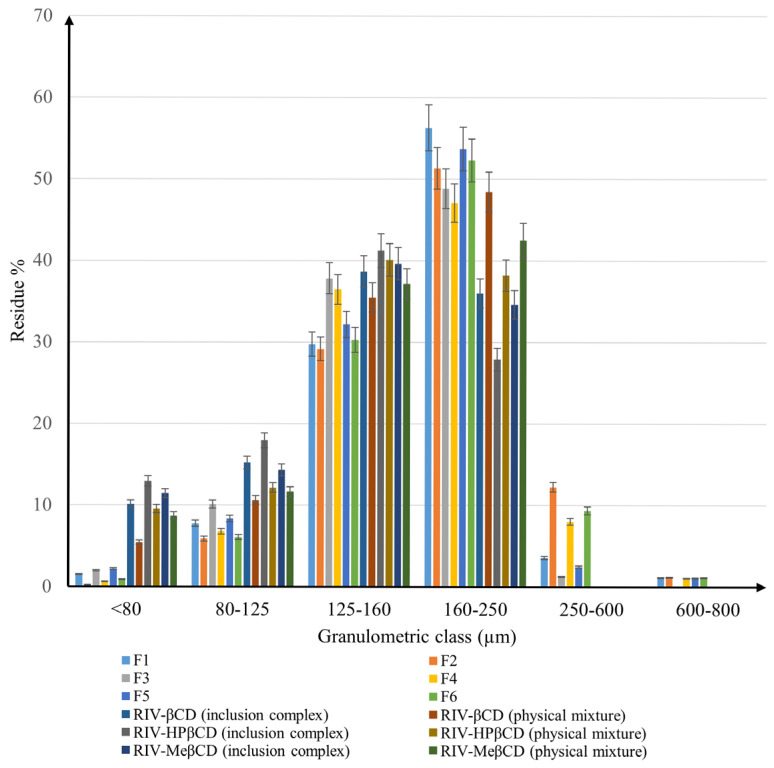
Granulometric analysis of binary systems and mixtures for direct compression.

Figure 6 shows the recorded histogram for the powders examined, in which the particle size distribution is shown according to granulometric classes (the formulations F1, F2, F3, F4, F5, and F6 are defined in the Section 3).

There is a clear difference between the samples in terms of the fineness of the powders. It is obvious that most of the particles of all materials have sizes in the range of 125–250 µm. According to Shekunov, B.Y. et al. [17], a narrow distribution of particle size provides the powders with good flowability and compressibility features. On the other hand, Wünsch I. et al. [18] stated that the resistance to compression increases with decreasing particle size, which is probably due to a significant contribution of the deformation mechanisms during compression.

Among binary systems, the differences are not very pronounced, but in all cases, the physical mixtures had a higher proportion of particles in the 160–250 µm range than the inclusion complexes, demonstrating the influence of the preparation method on particle size. While in the inclusion complexes, the percentage of particles with sizes between 160 and 250 µm varies between 27.88% in the case of RIV-HP-β-CD and 36% for the RIV-β-CD complex, for the physical mixtures it fluctuates between 38.2% (RIV-HP-β-CD) and 48.45% (RIV-β-CD). It should also be noted that no binary system has particles larger than 250 µm.

As for the inclusion compounds, all of them showed a higher percentage of particles with a size of 125–160 µm, while in the physical mixtures, the majority of particles were in the 160–250 µm granulometry class. Nevertheless, the RIV-HPβCD inclusion complex has the highest proportion (41.23%) of particles with a width of 125–160 µm compared to the RIV-βCD inclusion complex, which has 38.66% of particles in the same interval.

Concerning the powders for direct compression, the influence of the excipients added to the active ingredients is considerable. For F1–F6, a significant change in particle size distribution can be observed compared to the binary systems, with an important essential increase in particle size. In these cases, particles larger than 250 µm were observed. The difference between the materials containing inclusion complexes and those based on physical mixtures also remains as in the binary systems, with a higher amount of particles sized between 250–600 µm. Whereas F2, which is based on the RIV-β-CD physical mixture, has the highest percentage of particles between 250 and 600 µm (12.24%), F3, which contains RIV-HP-β-CD inclusion complex, has only 1.26% of the particles in the same class. The proportions of particles with sizes above 600 µm are negligible and are less likely to have an impact on the mechanical behavior of the materials.

However, the influence of the type of cyclodextrin used on complex formation was also noted. It can be seen that HP-β-CD leads to the smallest particle size, β-CD produces the largest amounts of coarser particles, while Me-β-CD has granulometric properties in between.

Due to the differences identified, it can be assumed that the materials will exhibit different flow and compression behavior.

The pharmacotechnical characteristics of the samples are shown in Table 2.

A key powder characteristic that has a significant impact on the uniformity and behavior of the mixture during compression as well as on the quality of the final tablets is the moisture content [19]. It was assumed that the samples would have a certain moisture content, as the active components were prepared using the freeze-drying technique with water. Koumbogle K et al. [20] explained that moisture transfer is mostly realized by vapor diffusion, as the pores in the hygroscopic zone are primarily filled with water vapor which condenses on the capillary meniscus.

Powders’ flow energy and cohesiveness rise as the water content increases [21]. This is because the two main forces between particles—the adhesion force and the frictional force—are influenced by the moisture content [22]. Liquid bridges that form as a result of moisture being adsorbed on the particles surface can lead to capillary and higher cohesive forces between particles [23]. As a result, lumping and agglomeration of powder may appear, which impairs flowability [24]. However, water molecules between the powder particles can serve as a lubricant, lowering particle friction and enhancing flowability [25].

As expected, the lyophilization inclusion complexes contain the highest amount of moisture, ranging from 4.03% for the RIV-β-CD inclusion complex to 4.78% for the RIV-MeβCD inclusion complex. There is a significant difference between the water content of the inclusion complexes and the corresponding physical mixtures or direct compression powders, with the freeze-dried powders containing double the amount of humidity. Among the CD types, all β-CD-based materials have the lowest moisture content, while the powders with Me-β-CD enclose the highest proportion of water.

Furthermore, a considerable difference was found between the mixtures for direct compression and the binary systems. These differences are due to the added excipients, which have a low water content and greatly reduce the moisture of the powders. The same pattern as for the binary systems was also found for the direct compression materials, with the β-CD-based powders having low moisture content and the Me-β-CD-based powders having the highest values.

Considering that the selected excipients are the same in all formulations, no individual influence can be detected, but it is clear that the type of CD and the preparation method of the binary system significantly influence the moisture content of the samples.

According to Chendo C. et al. [26], in tablet manufacturing, uneven flow, segregation or bridging are responsible for obtaining tablets with excessive mass variation or poor content uniformity. The presence of interparticle forces between small particles (usually less than 10 μm) and between small and larger particles determines the powder’s resistance to flow. These forces are caused by adhesion and cohesion forces that act between the surfaces of the different particles; this is called interactive mixing. Van der Waal’s, capillary, and electrostatic forces are the most frequent forces acting between the powder particles; the strength of these forces determines how the interactive mixture forms. When the adhesive forces are greater than the cohesive forces between the smaller particles, disagglomeration and preferential adhesion between the larger and smaller particles are energetically beneficial. This phenomenon is called cohesive–adhesive equilibrium.

The flowability increased significantly after addition of the excipients to the binary systems. It was found that the inclusion complexes formed by RIV with all CDs did not flow even when using the largest nozzle of 25 mm and the highest stirring speed of 25 rpm, proving the lack of flowability of these systems. This is due both to the CDs, which are known for their poor flow behavior [27], and to the freeze-drying process, which leads to amorphous powders [28]. The corresponding physical mixtures exhibited poor flowability, but the measurement could be performed even if it was done through a 25 mm nozzle with 25 rpm stirring. There is a noticeable difference between the mixtures depending on the CD type, with the RIV-βCD mixture having the fastest flow rate of 2.521 g/s and the RIV-Me-βCD mixture having the slowest flow rate of 2.112 g/s and a flow time 5 s longer than the RIV-βCD mixture.

In contrast, all six powder formulations for direct compression showed greatly improved flow performance, all flowing through the 15 mm nozzle without the need for stirring. Nevertheless, none of the samples has excellent flow behavior according to the European Pharmacopoeia criteria [29], but all have suitable flow behavior for direct compression process. As expected, the formulations based on the physical mixtures exhibited better flow rates than the materials containing lyophilized compounds, but this time the blends based on RIV-HP-βCD showed the highest flow rate of 3.896 g/s for the composed powder containing the RIV-HP-βCD inclusion complex and 4.054 g/s for the one including the RIV-HP-βCD physical mixture. As in the case of the binary systems, the RIV-Me-βCD-based blends exhibit the lowest flow profile, highlighting the influence of the CD type on the flowability of the materials.

Volumetric characteristics such as the Hausner ratio and the Carr index are also important for assessing flowability and predicting compaction or segregation problems. These properties are important parameters for assessing the compressibility potential of the materials, as they reflect the interactions between the particles that affect the flow behavior. These interactions are negligible for free-flowing powders and only lead to minor deviations between bulk and tapped density. In contrast, there is a clear difference between these densities for materials with poor flow properties, as the interactions between the particles are stronger. In this sense, an increase in bulk density is advantageous for die filling in the production of tablets, as voluminous mixtures improve the powder compression processing. In particular, the Hausner ratio indicates how likely it is that powders will consolidate under mechanical stress. How easily a powder compacts under mechanical stress is indicated by the Carr index [30,31].

The results of the volumetric analysis are consistent with the flowability findings, showing that the inclusion complexes have a “very, very poor flowability” [32], with CI above 37 and HR above 1.6. Among them, RIV-Me-β-CD has the highest values of 42.18 for CI and 1.72 for HR. Meantime, the values registered for the simple physical mixtures of RIV and CDs show poor flowability in the case of RIV-β-CD (CI is 25.96 and HR is 1.35) and very poor flowability in the case of RIV-HP-β-CD (CI is 31.52 and HR is 1.46) and RIV-Me-β-CD (CI is 31.74 and HR is 1.47). The results confirm that the flowing and compressing behaviors of the materials are influenced by both the type of CD used and the process used to obtain the binary systems.

The powders for direct compression showed better flowability and suitable compressibility compared to the binary systems, which can be attributed to the appropriate choice of excipients and especially the lubricant. Nevertheless, some differences between the formulations can be observed. The formulations containing the physical mixtures show better flowability than those containing the freeze-drying inclusion complexes, which is reflected in lower values for CI and HR. The decreasing order of flowability is maintained, RIV-HP-β-CD > RIV-β-CD > RIV-Me-β-CD. According to the specifications of the European Pharmacopoeia, only F4, which contains the physical mixture RIV-HP-β-CD, has “good flowability”, while F5, which contains the inclusion complex RIV-Me-β-CD, has only “passable flowability”. The other complex powders have “fair” flowability, but overall, all blends can be processed in the form of tablets by direct compression.

In pharmaceutical technology, the flow performance of powders is essential to maintaining uniformity and effectiveness across different manufacturing stages. For tablets to be mixed uniformly, filled precisely, and dosed accurately, powder materials must have predictable flow characteristics. Blends must have consistent flow properties to avoid problems like uneven dosage units, clogged machines, and ingredient segregation. These issues can have a direct impact on product quality and regulatory compliance. Irregular powder flow poses various risks, such as weight fluctuations, uneven content, and possible rejection of the batch. Furthermore, the flow behavior has a significant impact on the production scalability. In key processes such as blending or compression, materials must flow between equipment effectively without clogging or separating. To achieve a uniform weight and hardness during tablet compression, a steady flow into the dies is necessary. Products may become unusable if there are any interruptions at this point, e.g., due to inadequate flow resulting in fractured tablets. Therefore, it is essential to comprehend the flow characteristics of powders to guarantee both process effectiveness and product quality.

Chen FC et al. [33] demonstrated that the functional properties of the powders (e.g., compressibility, flowability, lubricant sensitivity, etc.) are determined by the fundamental properties (e.g., density, particle size, etc.) and that the powder preparation technologies have the greatest influence on the particle structure. The analysis of the studied powders led to the same conclusions, as there is a clear correlation between the flow and compressibility behavior of the samples and their particle size distribution, density and moisture content. It was also emphasized that the different preparation methods of the binary systems strongly influence the physical properties of the materials. It was found that the freeze-dried powders, which contain a higher moisture content and a higher proportion of small particles, exhibited significantly lower flowability than the physical mixtures in whose preparation no solvent was used. The same pattern is maintained after the addition of excipients for direct compression, but the physical properties are greatly improved in the final composed powders.

Qu L. et al. [34] proved that only a small amount of magnesium stearate can considerably improve the flowability of the materials and does not affect the disintegration behavior of the final tablets.

The lyophilized inclusion complexes with amorphous character exhibited a significantly lower bulk density than the crystalline physical mixtures, but the tapped density is much higher; compared to crystalline materials, amorphous powders are more susceptible to plastic deformation and have a higher compressibility [35]. Also, it is known that the freeze-drying process leads to the formation of loose aggregates and porous structures during the drying phase [36]. A low bulk density indicates a high densification tendency. It is caused by a highly porous structure and high particulate imperfections [37].

The positive influence of the selected excipients on the flowability and compressibility of the materials was also established. As Thoorens G et al. [38] found, microcrystalline cellulose increases the bulk density of the powders. According to Olorunsola EO et al. [39], the addition of microcrystalline cellulose is recommended for active ingredients with high moisture content in order to provide sufficient and efficient compressibility to the powder mix, as the presence of water negatively affects the compressibility of powders and the tensile strength of tablets. As Chitedze et al. [40] have shown, when microcrystalline cellulose is added to a powder with low flowability, the Hausner ratio, Carr index, and angle of repose decrease significantly, as the cohesion and compaction of the particles decrease and thus their porosity increases.

FlowLac^®^ 100 is also adequate for direct compression due to its good flowability and compaction qualities. It produces tablets with good mechanical strength, as well as faster disintegration and dissolution [41]. Nevertheless, it has appropriate deformability for direct compression, which is indicated by the Heckel yield pressure [42]. According to Al-Zoubi N et al. [43], with a suitable formulation design, it is likely to obtain a ready-to-compress blend even when starting from active ingredients with low pre-compression attributes.

### 2.5. Quality Control of the Tablets

**Figure 7:**
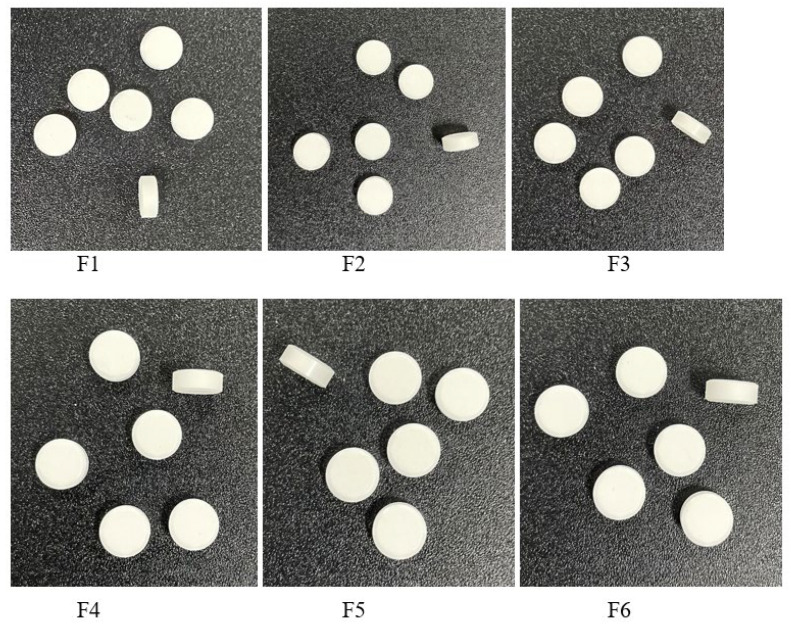
Tablets appearance. F1–F6 represent the code for the pharmaceutical formulations.

The tablets obtained have a white color, a round shape, a smooth surface, and a uniform appearance (Figure 7).

The pharmacotechnical characteristics of the six batches of tablets are shown in Table 3.

The tablets’ dimensions (thickness and diameter) and weight vary within narrow limits, which proves that the dies were filled uniformly due to the sufficient flowability of the materials. The compression process was also successfully conducted due to the appropriate adjustment of all parameters and the suitable compactability of the materials. Minor variability within and between batches shows that the type of cyclodextrin has no influence on the mass or size of the tablets. In all six formulations, the tablets have a weight of about 200 mg, a diameter of 10 mm, and a thickness of about 2.70 mm. These values demonstrate the correct choice of excipients and compression conditions and fulfil the requirements of the European Pharmacopoeia [29].

Achieving uniformity of size and mass ensures satisfactory dosage and uniformity of content of tablets. Hashmat D. et al. [44] state that powder properties, fluctuation in die filling and compression force have an influence on how uniformly the weight of the tablets varies. It is concluded that each of these elements was adequately determined and tablets with sufficient and within-limit properties were produced.

In contrast, the hardness varies greatly between the individual batches, which demonstrates the different compaction ability of the blends. The hardness varies between 67 N and 86 N, and there is also a strong variation between batches of the same RIV-CD system. Considering that all formulations contain the same excipients in the same amounts, this means that both the CD type and the process used to produce the binary system have a major influence on the plasticity and elasticity properties of the compounds. While in the βCD and Me-β-CD systems, the tablets with the physical mixture have a higher hardness value than those with the freeze-dried inclusion complexes; in the case of HP-β-CD, the F3 with freeze-dried active ingredient has a higher strength (84 N) than the F4 with the corresponding physical mixture (67 N). The highest value for hardness was registered for F2, containing the physical mixture RIV-β-CD (86 N), followed by F6 containing the physical mixture RIV-MeβCD. Although there are significant differences between the formulations, the mechanical resistance of all tablets is adequate in terms of both hardness and friability.

The friability of the tablets is more uniform between batches, but all values are within the compendial limit (<1.0%). The highest value was recorded for the MeβCD tablets, with F5 showing a friability of 0.10% and F6 showing a weight loss of 0.11%.

With regard to the time required for the tablets to completely disintegrate, there are major differences between the individual batches. It is clear that all formulations containing the inclusion complexes require a shorter disintegration time than the tablets containing the corresponding physical mixture. Nevertheless, the difference due to the type of CD is greater than the difference due to the technological process used to obtain the binary systems. The fastest disintegration was recorded for β-CD tablets, with a needed time of 35 s for F1 and 40 s for F2. This was followed by HP-β-CD-based tablets, with F3 taking 88 s and F4 97 s to disintegrate. Me-β-CD-based tablets had the slowest disintegration time, requiring 123 s for F5 and 145 s for F6 to decay.

Paul S. et al. [45] showed that the internal structure of the tablet, its mechanical strength, and its material properties can have an influence on its friability. Under the same compression force, tablets made of brittle material are often more friable than those made of plastic materials. At the same time Gong et al. [46] proved that a tablet’s diametrical deformation and its friability are related.

Also, Cabiscol R et al. [47] revealed that because of the stress gradients during compression process and the frictional and adhesive forces between the material and the die walls, powder densification during uniaxial compaction is controlled by several simultaneous processes occurring over a short period of time. This leads to the development of an anisotropy in density and stiffness in both the axial and radial directions.

Microcrystalline cellulose is known for its soft/plastic behavior, while lactose has a medium deformability [47]. The joint use of the two excipients in the studied tablet formulations resulted in a synergistic effect, ensuring adequate friability and good hardness at the same time. The mechanical strength of the tablet results from the combined interaction of mechanical interlocking, elasto-plastic deformation, and fragmentation during powder compression [48]. The tendency of acicular grades of microcrystalline cellulose to align perpendicular to the die axis during filling and tapping indicates their preferential orientation [49].

Wang J. et al. [50] have shown that increasing the amount of magnesium stearate in tablets formulations causes a noticeable delay in drug release and tablet disintegration due to diminished tablet wettability. Considering the excellent disintegration time achieved by all batches of tablets, it can be stated that the 1% magnesium stearate content ensures suitable flowability of the material and satisfactory pharmacotechnical properties of the tablets, which proves its appropriate selection. The results are consistent with the findings of Rojas J et al. [51], which show that the disintegration time of microcrystalline cellulose and sodium starch glycolate is not affected by magnesium stearate.

The disintegration behavior of tablets has a major impact on the performance of a drug. The disintegration process is particularly important in immediate release formulations. Sodium starch glycolate acts as a disintegrant mainly through a swelling mechanism, i.e., when its particles come into contact with water, their volume expands in all directions, increasing liquid penetration [52]. Considering the short disintegration time of the tablets containing RIV-CDs, it can be concluded that the type and concentration of the superdisintegrant were chosen correctly. Meanwhile, the CD type clearly has a great influence on the disintegration performance of the final tablets. Among them, βCD seems to have the highest disintegration activity.

The tablets’ disintegration behavior has a great impact on a drug’s performance. In the case of immediate-release dose formulations, the disintegration process is particularly important. Sodium starch glycolate acts as a disintegrant mainly through the swelling mechanism, i.e., when its particles come into contact with water, their volume expands in all directions, increasing liquid penetration [53]. Considering the short disintegration time of the tablets containing RIV-CDs, it can be concluded that the type and concentration of the superdisintegrant were chosen correctly. Meanwhile, the CD type clearly has a great influence on the disintegration performance of the final tablets. Among them, β-CD seems to have the highest disintegration activity.

**Figure 8:**
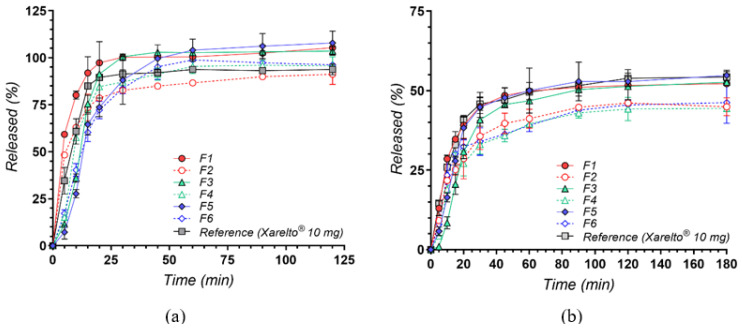
In vitro dissolution profiles of rivaroxaban from test formulations (F1–F6) compared with the reference product (Xarelto^®^ 10 mg) in (**a**) pH 4.5 sodium acetate buffer with 0.2% SDS and (**b**) pH 6.8 phosphate buffer. Results are presented as mean ± SD (*n* = 3).

The in vitro dissolution profiles of rivaroxaban (RIV) from six newly developed tablet formulations (F1–F6) compared with a commercial reference (Xarelto^®^ 10 mg, Bayer AG, Leverkusen, Germany) are shown in Figure 8. Two testing media were employed: pH 4.5 sodium acetate buffer containing 0.2% sodium dodecyl sulphate (SDS)—the compendial condition for 10 mg RIV tablets, which means that the sink conditions are met according to the compendial recommendations for the analysis—and pH 6.8 phosphate buffer without surfactants, designed to mimic near-neutral intestinal environments. This second medium is not under sink conditions, but has high biorelevance for the behavior and performance of the drug at the gastrointestinal level. As rivaroxaban exhibits limited aqueous solubility [54], both media and surfactant selection play pivotal roles in achieving reliable and physiologically relevant drug release data.

Under mildly acidic conditions with a surfactant, rivaroxaban’s limited solubility is substantially overcome, facilitating rapid drug release across all test formulations (Figure 8a). Among the cyclodextrin-based products, the complexes (F1, F3, F5) consistently achieved higher or faster release compared to their corresponding physical mixtures (F2, F4, F6), demonstrating the advantage of pre-formed inclusion complexes in promoting the drug’s wettability and reducing crystallinity. Notably, these differences were somewhat diminished over time, as SDS effectively maintained sink conditions [54]. The reference formulation (Xarelto^®^ 10 mg) also displayed rapid dissolution, bolstered by its specialized excipients (e.g., superdisintegrants, binders, and wetting agents) [55]. Notably, at the earliest time points under sink conditions, its dissolution percentages were marginally lower than those achieved by certain cyclodextrin-based complexes, likely reflecting the enhanced initial solubilization conferred by pre-formed drug–cyclodextrin inclusion.

In contrast, the absence of SDS in the pH 6.8 medium highlights the intrinsic solubility constraints of rivaroxaban [56]. Shifting to a near-neutral pH 6.8 buffer without surfactant (Figure 8b) highlights more pronounced differences in formulation performance, paralleling fasted-state conditions in the small intestine. Here, the influence of cyclodextrin complexation on drug release becomes more pronounced: the three pre-formed complexes (F1, F3, F5) maintained noticeably higher dissolution rates and extents than their physical mixture counterparts (F2, F4, F6). These improvements stem from enhanced molecular dispersion and minimized crystallinity afforded by the complexation process [57]. Overall, the commercial tablet maintained consistently strong performance; however, certain cyclodextrin-based complexes (particularly F1 and F3) reached similarly high dissolution values at later sampling points, with profiles nearly matching those of the reference product. These findings underscore the promise of such complexation strategies in enhancing rivaroxaban solubility, even under less favorable gastrointestinal conditions. Interestingly, after about 90 min, the dissolution profiles for all formulations reached a plateau, suggesting that the medium had approached saturation with respect to rivaroxaban. This behavior aligns with the drug’s reported solubility limit of approximately 6 µg/mL at pH 6.8 [54], which diminishes further differentiation among the tested formulations beyond this point.

In essence, performing dissolution studies in these two physiologically relevant media provides a more complete picture of the formulations’ behavior across varying pH ranges and levels of surfactant availability. Testing at pH 4.5 with SDS ensures a scenario that mirrors a more fed-like or surfactant-rich environment in the upper GI tract, where rapid dissolution can be critical for drugs with limited aqueous solubility. Meanwhile, the pH 6.8 condition approximates the fasted-state small intestine, providing insight into how each formulation might perform once the dosage form transits beyond the acidic region of the GI tract. By examining both dissolution setups, it becomes evident that cyclodextrin-based complexes offer a distinct advantage in solubilizing rivaroxaban, particularly when exogenous surfactants are absent. These findings support the continued exploration of cyclodextrin approaches to enhance the bioavailability of rivaroxaban tablets and can guide further optimization of formulation parameters aimed at achieving both rapid onset and reliable drug release under physiologically relevant conditions.

The formulations containing CD exhibited release profiles comparable to Xarelto^®^ 10 mg, achieving similar dissolution performance without the need for SLS in the tablet composition, thereby offering a potentially improved safety profile.

## 3. Materials and Methods

### 3.1. Materials

Labormed-Pharma SA kindly donated micronized RIV (Form I) produced by Neuland Laboratories Limited. Global Holding Group Co., Ltd. (Ningbo, China) supplied the three cyclodextrins (β-CD, HP-β-CD and Me-β-CD). Avicel^®^ PH 102 was purchased from International Flavors and Fragrances Inc. IFF, New York, NY, USA and Flowlac^®^ 100 was provided from Meggle GmbH & Co. KG, Wasserburg am Inn, Germany. EXPLOTAB^®^ was produced by JRS PHARMA GmbH & Co. KG, Rosenberg, Germany and LIGAMED^®^ MF-2-V by Peter Graven NV, Gelderland, The Netherlands. Each chemical and solvent used had the grade of an analytical reagent. The materials were weighed using a Mettler Toledo AT261 balance (Mettler Toledo, Greifensee, Switzerland) with a sensitivity of 0.01 mg.

Acetonitrile (gradient grade, suitable for HPLC) was obtained from Merck KGaA (Darmstadt, Germany). Formic acid (99.0+%, Optima™ LC/MS Grade) and sodium hydroxide (extra pure, 50 wt% solution in water) were purchased from Fisher Chemical (Thermo Fisher Scientific, Waltham, MA, USA). Ultrapure water (18.2 MΩ·cm at 25 °C) was generated using a Milli-Q EQ 7008 purification system (Merck Millipore, Burlington, MA, USA). Additional reagents, including sodium acetate, acetic acid, sodium dodecyl sulphate, and potassium phosphate monobasic, were supplied by Merck KGaA (Darmstadt, Germany).

### 3.2. Solubility Studies

#### Phase-Solubility Diagrams

Three solutions of the selected cyclodextrins (β-CD, Me-β-CD and HP-β-CD) were initially prepared as follows:

(1) 0.0145 M solution of β-CD by dissolving 0.342 g of β-CD in 20 mL of distilled water;
(2) 0.0196 M solution of Me-β-CD by dissolving 0.513 g of Me-β-CD in 20 mL of distilled water;
(3) 0.1006 M solution of HP-β-CD by dissolving 2.061 g of HP-β-CD in 15 mL of distilled water.

The rapid dissolution of cyclodextrins in distilled water was achieved by stirring at 750 rpm at room temperature for a few minutes using a Heidolph MR 3001 K magnetic stirrer (Schwabach, Germany). Amounts of rivaroxaban (approximately 10 mg) were added to five test tubes for β-CD, Me-β-CD, and HP-β-CD, each containing increasing volumes (1, 2, 3, 4, and 5 mL) of the prepared cyclodextrins solutions. These were then brought to a total volume of 5 mL with distilled water.

The tubes were stirred at 750 rpm for 6 h at room temperature (25 ± 2 °C) using a Heidolph MR 3001 K magnetic stirrer (Schwabach, Germany). The samples were filtered through a 0.45 μm nylon membrane filter (Whatman^®^ Puradisc™, Dreieich, Germany). The absorbance was measured at 248 nm using UV-Vis Perkin-Elmer Lambda 35 spectrophotometer (Shelton, CT, USA). All experiments were conducted in duplicate.

Based on the Higuchi and Connors phase-solubility diagrams [58], the apparent stability constant (*K_st_*) was calculated assuming the formation of a 1:1 stoichiometric inclusion complex, using the following equation:

(1) $$K_{st}=\frac{slope}{S_{0}\left(1-slope\right)}$$

where *K_st_* is the apparent stability constant of the 1:1 inclusion complex, *S*_0_ is the intrinsic solubility of rivaroxaban in the absence of cyclodextrins (estimated from the intercept of the phase-solubility curve)

The slope was determined from the initial linear portion of the rivaroxaban phase-solubility diagram plotted against each cyclodextrin concentration. For calibration, a reference solution was prepared by dissolving 2.5 mg of rivaroxaban in 500 mL of distilled water. The average absorbance was used to determine the conversion factor according to the equation:

(2) c(M)=f×Abs

where *c* is the molar concentration, *f* is the calibration factor, and *Abs* is the measured absorbance.

### 3.3. Methods

#### 3.3.1. Synthesis

##### Preparation of the RIV-Cyclodextrins Binary Systems

Six different binary systems were prepared while maintaining the same molar ratio of 1:1 between RIV and CD. Three of them were the inclusion complexes of RIV in β-CD, HP-β-CD, and Me-β-CD cavities, and the other three were simple physical mixtures that served as references for the characterization studies of the guest–host compounds.

##### Preparation of the RIV-Cyclodextrin Inclusion Complexes

Each CD was dissolved in an appropriate amount of water, and the RIV was dissolved in acetone within a sealed glass container. After gradual addition of the aqueous CD solutions to the RIV solution, the resulting suspensions were stirred for eight hours at room temperature using a Heidolph MR 3001 K magnetic stirrer at 850 rpm. The samples were frozen and then freeze-dried for 15 h at −60 °C in a lyophilizer CoolSafe Basic and Pro, produced by Labogene A/S, Allerod, Denmark [59,60].

##### Preparation of the RIV-Cyclodextrins Physical Mixtures

The physical mixtures were prepared in solid state by simply mixing the RIV with the CDs for 2 min at room temperature [58].

#### 3.3.2. Physicochemical Characterization of the Binary Systems

The infrared spectroscopic measurements were performed using the NICOLET 6700 FT-IR spectrophotometer (Thermo Electron Corporation, Waltham, MA, USA), based on Fourier transform infrared (FT-IR) spectroscopy, in transmission mode within the 400–4000 cm^−1^ range, at a resolution of 4 cm^−1^. The spectra were recorded on a thin, transparent KBr pellet (20 mg/cm^2^) containing approximately 0.5% of the sample. The pellets were prepared by compacting and vacuum-pressing a homogeneous mixture obtained by grinding 1 mg of the substance with 200 mg of KBr.

The thermal experiments were performed on a NETZSCH STA 449 F3 Jupiter (Selb, Germany), within 25–600 °C. The DTA and TG curves were recorded under a nitrogen atmosphere with 20 mL/min flow rates and at a heating rate of 10 °C/min NETZSCH Proteus—Thermal Analysis—version 5.2.1 software was used for processing the DTA-TG experiment.

Room temperature X-ray diffraction measurements were performed on a Bruker D8 Advance diffractometer using Ni-filtered Cu-Kα radiation (λ = 1.5418 Å). The X-ray tube was operated at 40 kV and 40 mA. On the incident beam side, motorized slits with 0.25 mm opening and a 2.5° soller slit were used, and on the diffracted beam side, motorized slits with 5 mm opening were mounted on LYNXEYE XE-T detector operated in 1D mode and high-resolution option. The X-ray diffraction patterns were recorded in the 5–60° 2θ range, with a step size of 0.02° and a counting time of 0.2 s/step.

Morphology of the raw materials and complexes was determined using a scanning electron microscopy (Tescan Vega LMU, Brno, Czech Republic) operated in a low vacuum of 20 Pa and at an accelerating voltage of 10 KV.

#### 3.3.3. Precompression Studies for the Tablets Containing RIV—CD Binary Systems

Considering all its advantages—especially the absence of moisture throughout the process, which protects the ingredients—direct compression is the preferred manufacturing process for the fast-release tablets to be developed in this study. To ensure that the final tablets have suitable pharmacotechnical and stability properties, it is important to produce a direct compression blend with high flowability and compressibility [61]. Since the lyophilization complex is amorphous, it is particularly difficult to prepare blends with the right physical properties for direct compression technology, and it requires careful selection of excipients.

##### Formulations of the Materials for Direct Compression

The amounts of the individual ingredients were calculated to obtain tablets with a total weight of 200 mg, corresponding to a concentration of 10 mg RIV. Six series of composed powders were prepared, three of which contained the inclusion complexes as active ingredients and the other three the simple physical mixtures. Table 4 displays the formulations that were selected for each binary system.

The influence of both the binary systems used as active ingredients and the excipients on the materials’ properties was investigated. For all formulations, Avicel^®^ PH 102 (microcrystalline cellulose)(FMC Corporation, Pennsylvania, USA) and Flowlac^®^ 100 (spray-dried lactose)(MEGGLE Group, Wasserburg, Germany) were selected for their consistent filling, binding, and disintegrating properties, but mainly for their effective flowability and compressibility, mandatory attributes for the direct compression process, especially for amorphous active ingredients characterized by poor flow properties [62,63]. Ligamed^®^ MF-2-V (magnesium stearate) (MEGGLE Group, Wasserburg, Germany) was used for its gliding properties, while Explotab^®^ (sodium starch glycolate) (JRS Pharma, Rosenberg, Germany) was selected as a superdisintegrant [64].

##### Preparation of the Direct Compression Blends

After passing through a 20-mesh sieve, the ingredients were weighed in the specified quantities. In a CMP 12 Plexiglas cube mixer from Pharmag GmbH, Klipphausen, Germany; all ingredients (with the exception of the magnesium stearate) were mixed at a speed of 30 rpm for 20 min at room temperature. Finally, magnesium stearate was added under the same conditions and stirred for a further two minutes.

##### Pharmacotechnical Analysis of the Materials

All analyses were performed for all binary systems (inclusion complexes and physical mixtures) and all direct compression mixtures.

Fineness was investigated by analytical sieving with a CISA sieving shaker Mod. RP 10, manufactured by Cisa Cedaceria Industrial in Barcelona, Spain. After arranging the sieves in decreasing order of fineness and pouring 50 g of each powder onto the top sieve, the system was shaken for ten minutes at an amplitude of 700 rpm. The retained powder from each sieve was collected and weighed.

Moisture content was indicated by the loss on drying measured by the thermogravimetric method using a Mettler-Toledo GmbH, Greifensee, Switzerland, HR 73 Mettler Toledo halogen humidity analyzer.

Flowability was expressed by the flow rate, flow time, and angle of repose registered when 60 g of each sample was passed through a standardized diameter orifice. An Automated Powder and Granulate Testing System PTG-S3 from Pharma Test Apparatebau GmbH in Hainburg, Germany was used for test.

Compressibility was assessed by calculating the bulk and tapped density, the Hausner ratio (HR), and the Carr Index (CI). For the analysis, the cylinder of the Vankel Tap Density Tester, manufactured by Vankel Industries Inc., Cary, NC, USA, was filled with 30 g of each sample. First, the bulk volume was read, then the tapped volume after applying 500 mechanical shocks. Finally, the following formulas are used to calculate HR and CI:

(3) $$HR=\frac{\rho_{tapped}}{\rho_{bulk}}$$

(4) $$CI\left(\%\right)=100\times \frac{\left(\rho_{tapped}-\rho_{bulk}\right)}{\rho_{tapped}}$$

where *ρ_tapped_* is the tapped bulk density of the blend (g/cm3) and *ρ_bulk_* is the loose bulk density of the material (g/cm^3^).

An HR value below 1.25 indicates that the powder is free-flowing, and a CI value below 10 means that the material has excellent flowability and compressibility [65].

#### 3.3.4. Development and Manufacturing of the Tablets

##### Formulation of the Oral Tablets

Based on the results of the preformulation trials, all six materials designed for direct compression showed pharmacotechnical properties suitable for processing into tablets. Table 5 displays the formulations of the oral tablets.

##### Manufacturing Process of Tablets

In a single-post eccentric tablet press, Erweka EP-1 from Erweka, Langen, Germany, the previously obtained materials were compressed with different compression forces as required (5 kN for F1, F4-F6; 9 kN for F2; 6 kN for F3). To achieve the optimal tablet characteristics, such as appropriate hardness, uniform mass, and consistent dimensions, across all batches, initially, a primer compression procedure for each formulation was conducted. During this step, the tablets were initially compressed with a standard force, and the resulting tablets were measured for thickness, diameter, weight, and hardness. This allowed for the identification of the necessary adjustments to the compression force to ensure that each batch met the required standards. The variation in compression forces across the different formulations was essential to ensure the uniformity of the tablets and to optimize their pharmacotechnical properties, which are influenced by the type of cyclodextrin and the degree of complexation. The machine was configured for the production of 200 mg tablets using 10 mm flat punches.

#### 3.3.5. Quality Control of the Tablets

##### Organoleptic Properties

The tablets’ overall appearance was examined as reported by the European Pharmacopoeia [29].

##### Dimensions (Diameter and Thickness)

A VK 200 tablet hardness tester from Vanderkamp, New York, NY, USA, was used to test the thickness and diameter of ten tablets of each formulation.

##### Mass Uniformity

The 20 tablets of each formulation were weighed independently, and the average weight was calculated [29].

##### Hardness

The hardness was measured with the VK 200 tablet hardness tester. It is expressed as the force required to crush the tablets positioned between the two anvils of the device. Ten tablets from each batch were analyzed.

##### Friability

It was determined using the Vankel friabilator (Vanderkamp, New York, NY, USA) on ten tablets from each series. After weighing, the tablets were introduced in the drums of the device and spun at 25 rpm for five minutes. After dedusting, the tablets were weighed again to determine the mass loss. 1.0% is the upper limit according to compendial standards [29].

##### In Vitro Disintegration Time

The disintegration behavior was evaluated on six tablets of each formulation in distilled water at 37 ± 0.5 °C according to the compendial norms [29]. An Erweka DT 3 device, manufactured by Erweka^®^ GmbH in Langen, Germany, was used to measure the time in seconds required for total disintegration.

#### 3.3.6. In Vitro Drug Release Studies

Drug release profiles of the 10 mg rivaroxaban tablets were evaluated using a Vision G2 Classic 6 Dissolution Tester (Teledyne Hanson, Chatsworth, CA, USA), configured with USP Apparatus II (paddles). Dissolutions were conducted at 37.0 ± 0.5 °C and 75 rpm, in line with the USP guidelines for rivaroxaban tablets [66]. Each vessel contained 900 mL of dissolution medium, and two different media were assessed: 0.022 M sodium acetate buffer at pH 4.5 containing 0.2% sodium dodecyl sulphate (the compendial medium recommended for 10 mg rivaroxaban tablets), and a 0.05 M phosphate buffer at pH 6.8 without surfactants.

Aliquots of 1.5 ± 0.1 mL were withdrawn at 5, 10, 15, 20, 30, 45, 60, 90, 120, and 180 min (the 180-min sampling was applicable only for the pH 6.8 buffer). After each withdrawal, an equivalent volume of fresh, preheated medium was added to maintain sink conditions. The collected samples were filtered through a 0.45 μm polyethersulfone membrane prior to analysis. All dissolution runs were performed in triplicate.

For a clearer assessment of the drug release performance, the same dissolution tests were also performed on a commercially available reference product, Xarelto^®^ 10 mg (Bayer AG, Leverkusen, Germany).

#### 3.3.7. HPLC Analysis

Rivaroxaban quantification followed a validated reversed-phase HPLC procedure adapted from a previously reported method [67]. Chromatographic separations were carried out on a Jasco 4000 Series HPLC system (JASCO Corporation, Tokyo, Japan), using a 100 × 3 mm Kinetex^®^ C18 column (2.6 µm particle size, Phenomenex, Torrance, CA, USA) maintained at 45 °C. The mobile phase comprised 0.1% formic acid (A) and acetonitrile (B), mixed at a 62:38 (*v*/*v*) ratio. UV detection was set to 250 nm. The HPLC assay was validated in accordance with current ICH guidelines [68], ensuring linearity, accuracy, precision, specificity, and sensitivity. Calibration curves were generated using standard solutions of rivaroxaban over a concentration range of 0.156–20 µg/mL, enabling accurate determination of the dissolved drug fraction in each sample.

## 4. Conclusions

The results of the present study highlight the significant potential of using cyclodextrin inclusion complexes and the lyophilization technique as an approach to enhance the solubility and dissolution properties of rivaroxaban, a poorly water-soluble drug. The successful formation of inclusion complexes, as confirmed by physicochemical methods such as FTIR, SEM, XRD, and TGA analyses, indicates that the encapsulation of rivaroxaban within the cyclodextrin cavity efficiently modifies its crystalline nature, thereby improving its wettability and dissolution rate. All three cyclodextrin derivatives used in this work demonstrated the considerable enhancement in solubility and stability of the rivaroxaban complex. This was reflected in both the dissolution profiles, where lyophilized formulations (F1, F3, F5) exhibited a faster and more complete release compared to physical mixtures, as well as in the physicochemical characterization, which showed significant improvements in thermal stability. Additionally, the pharmacotechnical properties of the lyophilized formulations, including suitable flowability, compressibility, and bulk density, further confirm the possibility of these complexes for incorporation into oral dosage forms, such as tablets and capsules. The results suggest that this formulation approach could be applied to other poorly soluble drugs, thus addressing one of the serious challenges in drug formulation. In conclusion, the combination of cyclodextrin complexation and lyophilization technique offers a promising and effective method for increasing the solubility of rivaroxaban. These results give significant insights into the development of improved oral formulations, with the potential to enhance the efficacy of rivaroxaban and similar drugs with poor aqueous solubility.

## Figures and Tables

**Table 1:** Parameters for the phase-solubility diagrams of the inclusion complex formed between rivaroxaban and each cyclodextrin.

System	Slope	Intrinsic Solubility (M)	Stability Constant (M^−1^)
rivaroxaban–β-CD	0.004	0.000008	251
rivaroxaban–Me-β-CD	0.0046	0.00001	289
rivaroxaban–HP-β-CD	0.0103	0.00003	650

**Table 2:** The pharmacotechnical parameters of the studied samples.

Formulation Code	Parameter							
	Moisture Content (%)	Flow Time (s) *	Angle of Repose (θ Degrees)	Flow Rate (g/s)	Bulk Density (g/mL)	Tapped Density (g/mL)	Carr Index (CI) (%)	Hausner’s Ratio (HR)
RIV-β-CD (inclusion complex)	4.03 ± 0.94	-*	-*	-*	0.258	0.427	39.57	1.65
RIV-β-CD (physical mixture)	2.14 ± 0.55	23.8 ± 0.27 *	36.14 ± 2.45 *	2.521 *	0.385	0.520	25.96	1.35
RIV-HP-β-CD (inclusion complex)	4.25 ± 0.79	-*	-*	-*	0.233	0.375	37.86	1.60
RIV-HP-β-CD (physical mixture)	2.63 ± 0.81	24.6 ± 0.38 *	37.27 ± 2.16 *	2.439 *	0.341	0.498	31.52	1.46
RIV-Me-β-CD (inclusion complex)	4.78 ± 0.83	-*	-*	-*	0.222	0.384	42.18	1.72
RIV-Me-β-CD (physical mixture)	2.97 ± 0.66	28.4 ± 0.15 *	39.09 ± 2.75 *	2.112 *	0.316	0.463	31.74	1.47
F1	1.86 ± 0.43	17.2 ± 0.25 **	29.11 ± 1.86 **	3.488 **	0.461	0.577	20.10	1.25
F2	1.47 ± 0.52	16.9 ± 0.47 **	28.82 ± 1.39 **	3.550 **	0.554	0.681	18.64	1.22
F3	2.39 ± 0.88	15.4 ± 0.28 **	27.66 ± 1.02 **	3.896 **	0.453	0.542	16.42	1.19
F4	1.98 ± 0.84	14.8 ± 0.31 **	27.08 ± 0.84 **	4.054 **	0.446	0.521	14.39	1.16
F5	2.61 ± 0.76	18.8 ± 0.23 **	30.15 ± 2.28 **	3.191 **	0.457	0.596	23.32	1.30
F6	2.14 ± 0.65	18.1 ± 0.44 **	29.79 ± 2.12 **	3.314 **	0.494	0.617	19.93	1.24

* 25 rpm stirring, nozzle: 25 mm; ** no stirring, nozzle: 15 mm.

**Table 3:** Quality characteristics of the tablets.

Parameter	Formulation Code					
	F1	F2	F3	F4	F5	F6
Thickness (mm)	2.71 ± 0.09	2.67 ± 0.11	2.70 ± 0.23	2.71 ± 0.34	2.69 ± 0.14	2.69 ± 0.36
Diameter (mm)	10 ± 0.25	10 ± 0.82	10 ± 0.19	10 ± 0.44	10 ± 0.30	10 ± 0.41
Mass uniformity (mg)	200 ± 1.68	199 ± 2.07	201 ± 1.05	200 ± 2.73	199 ± 1.54	200 ± 2.30
Hardness (N)	70 ± 2.85	86 ± 3.18	84 ± 3.67	67 ± 2.04	78 ± 3.09	85 ± 3.77
Friability (%)	0.05 ± 0.01	0.07 ± 0.03	0.07 ± 0.04	0.04 ± 0.02	0.10 ± 0.04	0.11 ± 0.03
In vitro disintegration time (seconds)	35	40	88	97	123	145

**Table 4:** The composition of the direct compression materials.

Ingredients	Formulation/Amount (%)					
	F1	F2	F3	F4	F5	F6
RIV-β-CD (lyophilized inclusion complex)	18	-	-	-	-	-
RIV-β-CD (physical mixture)	-	18	-	-	-	-
RIV-HP-β-CD (lyophilized inclusion complex)	-	-	22.50	-	-	-
RIV-HP-β-CD (physical mixture)	-	-	-	22.50	-	-
RIV-Me-β-CD (lyophilized inclusion complex)	-	-	-	-	20	-
RIV-Me-β-CD (physical mixture)	-	-	-	-	-	20
Avicel^®^ PH 102 –microcrystalline cellulose	40	40	37.75	37.75	39	39
Flowlac^®^ 100—spray-dried lactose	40	40	37.75	37.75	39	39
EXPLOTAB^®^—Sodium starch glycolate	1.00	1.00	1.00	1.00	1.00	1.00
LIGAMED^®^ MF-2-V—Magnesium stearate	1.00	1.00	1.00	1.00	1.00	1.00

**Table 5:** The formulations of the tablets containing RIV-CDs inclusion complexes and physical mixtures.

Ingredients	Quantity mg/Tablet						Role in Formulation
	F1	F2	F3	F4	F5	F6	
RIV-β-CD inclusion complex (1:1)	36	-	-	-	-	-	Active ingredient
RIV-β-CD physical mixture (1:1)	-	36	-	-	-	-	Active ingredient
RIV-HP-β-CD inclusion complex (1:1)	-	-	45	-	-	-	Active ingredient
RIV-HP-β-CD physical mixture (1:1)	-	-	-	45	-	-	Active ingredient
RIV-Me-β-CD inclusion complex (1:1)	-	-	-	-	40	-	Active ingredient
RIV-Me-β-CD physical mixture (1:1)	-	-	-	-	-	40	Active ingredient
Avicel^®^ PH 102 –microcrystalline cellulose	80	80	75.5	75.5	78	78	Filler Binder
Flowlac^®^ 100—spray-dried lactose	80	80	75.5	75.5	78	78	Filler Binder
EXPLOTAB^®^—Sodium starch glycolate	2	2	2	2	2	2	Superdisintegrant
LIGAMED^®^ MF-2-V—Magnesium stearate	2	2	2	2	2	2	Glidant
**TOTAL**	**200**	**200**	**200**	**200**	**200**	**200**	

## Data Availability

Data are contained within the article.
